# Body mass index and risk of cancer in young women

**DOI:** 10.1038/s41598-024-56899-1

**Published:** 2024-03-15

**Authors:** Pigi Dikaiou, Jon Edqvist, Jesper Lagergren, Martin Adiels, Lena Björck, Annika Rosengren

**Affiliations:** 1 Department of Molecular and Clinical Medicine, Sahlgrenska Academy, University of Gothenburg, Gothenburg, Sweden; 2 Sahlgrenska University Hospital/Sahlgrenska Hospital, Gothenburg, Sweden; 3 Sahlgrenska University Hospital/Östra Hospital, Gothenburg, Sweden; 4 Upper Gastrointestinal Surgery, Department of Molecular Medicine and Surgery, Karolinska Institutet, Karolinska University Hospital, Stockholm, Sweden; 5 School of Cancer and Pharmaceutical Sciences, King’s College London, London, UK; 6 Health Metrics Unit, Sahlgrenska Academy, University of Gothenburg, Gothenburg, Sweden; 7 Institute of Medicine, Department of Molecular and Clinical Medicine, Sahlgrenska Academy, University of Gothenburg Hospital/Östra, Diagnosvägen 11, 416 50 Gothenburg, Sweden

**Keywords:** Obesity, Cancer, Epidemiology

## Abstract

It is unclear how increasing body mass index (BMI) influences risk of cancer in young women. We used data from the Medical Birth, Patient and Cause of Death registers collected between 1982 and 2014 to determine the risk of obesity-related cancer types, breast cancer, all cancer and cancer-related death in relation to BMI in 1,386,725 women, aged between 18 and 45 years, in Sweden. During a median follow-up of 16.3 years (IQR 7.7–23.5), 9808 women developed cancer. The hazard ratio (HR) of endometrial and ovarian cancer increased with higher BMI from 1.08 (95% CI 0.93–1.24) and 1.08 (95% CI 0.96–1.21) among women with BMI 22.5–< 25 to 2.33 (95% CI 1.92–2.83) and 1.48 (95% CI 1.24–1.77), respectively, among women with BMI ≥ 30. There were linear and positive associations between BMI and incident cancer in the ovary, colon, endometrium, pancreas, rectum, gallbladder, esophageal cancer and renal cell carcinoma, as well as death from obesity-related cancer forms. In conclusion, we found that elevated BMI in young women linearly associated with several obesity-related cancer forms, including death from these cancers.

## Introduction

In Sweden, as in many high-income countries, cancer is the leading cause of death in young and middle-aged adults^1^. In the US, incidence of cancer in adolescents and young adults increased by almost 30% between 1973 and 2015^2^. Many cancers in young adult life are linked to lifestyle related risk factors^3^, and there is convincing evidence on the role of obesity in the development of cancer^4^, which could contribute to the increasing rates of cancer in the young. Mortality from cancer is higher among men than women, except for the age group 35 to 59 years, where mortality rates are higher in women, when the most common cancers are cancer of the breast and female genital organs^5^.

Obesity is associated with an increased risk not only of type 2 diabetes and cardiovascular disease^6,7^, but also of several types of cancer, including cancer of the esophagus, colon, rectum, kidney, pancreas, gallbladder, liver and specific to women, postmenopausal breast and endometrial cancer^8–10^. A high body mass index early, compared to later, in life has been proposed to be more strongly associated with several adult disorders, indicating that the current presence of many million young people with overweight or obesity may lead to a marked increase in several types of cancer in the near future and during decades to come^11^. Furthermore, reduced cancer incidence and mortality after weight loss have been reported among individuals with overweight and obesity^12^.

Globally, the age-standardized prevalence of obesity in women increased from 6.4 to 14.9% from 1975 to 2014 and is estimated to reach 21% by 2025^13^. In Sweden the prevalence of overweight and obesity in women of child-bearing age is currently almost 40% and 10%, respectively^14^. Because cancer is comparatively rare in younger persons, few studies have been adequately powered to investigate obesity and cancer risk in young women, and in particular specific cancer types. The Swedish Medical Birth registry contains data on height and weight in early pregnancy. In the present study, we used this information to investigate the relation between BMI in young women and risk of developing obesity-related cancer types, all cancer and cancer-related death.

## Methods

### Study population

For the purpose of the present study, we identified a cohort of all women, in their first or second pregnancy, aged between 18 and 45 years registered in the nationwide Swedish Medical Birth Register between January 1, 1982 and December 31, 2014 in Sweden. Women were mainly included in the study at the date of their first antenatal visit, but to enhance statistical power, women with incomplete data at first registration were allowed to be incuded when they were pregnant with their second child.

### Data collection

The Medical Birth Register includes information on age, height, weight, parity, tobacco smoking status, diabetes (type of diabetes not specified) and hypertension from all first antenatal visits, which as a rule take place within 6–12 weeks of gestation (in 90% of all pregnant women in Sweden) from 1973 onwards (with the exception of 1990 and 1991 when no weight data were recorded) with a coverage of 99%^15,16^. Height has been recorded at the first visit from 1992 onwards, whereas height between 1982 and 1992 was obtained from records on weight and height kept by the midwife at the delivery. Measured weight at the first antenatal visit has been registered since 1992 and was used as a proxy for prepregnancy weight in the present study. Between 1982 and 1989, early pregnancy weight was not recorded but was calculated from weight at delivery and weight gain. Weight at delivery was recorded using two digits (e.g. weight ≥ 100 kg was recorded as 99 kg). Valid information on height and weight was recorded in approximately 80% and 70% of cases, respectively. Over the period there was an increase in the prevalence of overweight and obesity; however a visual inspection of annual body weight deciles showed a sudden and larger than expected increase in body weight between 1989 and 1992. Because of this increase, the weights from 1982 to 1989 were adjusted by estimating annual weight increase within deciles from 1992 to 2003, which generated a nearly linear result^17^.

### Follow-up

The national healthcare system in Sweden provides tax-paid care to all residents. Hospitals and hospital-based outpatient clinics are required to report principal and contributory diagnoses to the Swedish National Patient Register and the Cause of Death Register. Data from the Medical Birth, Patient, and Cause of Death Registries and the Longitudinal Integration Database for Health Insurance and Labor Market Studies (LISA) were linked using the Swedish personal identity numbers. LISA was used to obtain information about educational level.

### Definitions

Diagnoses from hospital healthcare visits or discharges are registered in the Patient Register according to the International Classification of Diseases (ICD; ICD-8 used until 1986, ICD-9 1987–1996, ICD-10 from 1997) and information on cause- specific deaths was obtained from the Cause of Death Register (Supplementary Table 1 and Supplementary Table 2). The International Agency for research on Cancer (IARC) has identified 13 types of cancer associated with obesity^8^. We included esophagus cancer, thyroid, gallbladder, stomach, liver, pancreas, endometrial, ovarian, multiple myeloma, colorectal cancer and renal cell carcinoma. Breast cancer the most common form within the age range of the present study was not included as obesity-related cancer as long as only post-menopausal breast cancer was specified as obesity-related according to the IARC, and by definition, all women in the present study would have been premenopausal at baseline. Registrations of brain cancer in the Patient Register do not specify meningioma, another cancer that was specified as obesity-related by the IARC.

Cancer was defined as occurring in any patient as a primary or secondary hospital discharge diagnosis or death certificate with at least 1 cancer ICD code. Information on the following baseline comorbidities was obtained from the Patient Register: hypertension, diabetes, atrial fibrillation, chronic kidney disease, congenital heart disease, and valvulopathy. Information on hypertension and diabetes was also obtained from the Medical Birth Register (self-reported) and the subjects were assumed to have these comorbidities if it was recorded either as self-reported or in the Patient Register.

### Exposure

BMI was calculated by dividing body weight in kilograms by the square of height in meters (kg/m^2^). We categorized BMI into 6 groups: < 18.5 (underweight), 18.5–< 20 (lean), 20.0–< 22.5 (low normal), 22.5–< 25.0 (high normal), 25.0–< 30 (overweight), and BMI ≥ 30 (obesity). Additionally, models in which BMI was modelled as a linear term starting at BMI 20 were calculated (HR per 5 BMI units) as well as models in which BMI were modelled with cubic splines.

### Ethics statement

The present study was approved by the Regional Ethical Review Board in Gothenburg, Sweden (DNR: 103-15). Because the data are coded (pseudonymized) inform consent was waived by the Regional Ethical Review Board in Gothenburg for this study. The present study conforms to the principles outlined in the Declaration of Helsinki. All methods were carried out in accordance with relevant guidelines and regulations.

### Statistical analysis

Time to event was calculated separately for each cancer diagnosis at the time from inclusion to death or end of follow up (coded as no event), or first occurrence of the specific cancer diagnosis (coded as event). Compound measure of obesity related cancers was calculated as the time to first event.

Cox proportional hazard regression models ware used to calculate cause specific hazard ratios (HR) and 95% confidence intervals. Three models were developed; model 1 was adjusted for year of pregnancy (5 groups of equal size), and parity, model 2 was further adjusted for diabetes and hypertension at baseline and model 3 was further adjusted for smoking and education at baseline. No adjustment for new diagnoses of diabetes during follow-up was done given the strong association between BMI and diabetes type 2 where adjustment for this disorder would lead to underestimation of the true effect of an elevated BMI. Age was used as scale for the Cox regression modeling. The proportional hazards assumption was tested using methods based on weighted residuals^18^. For many outcomes, year of pregnancy did not fulfill the proportional hazards assumption. To make models comparable, all models were stratified by year (5 groups of equal size) and by parity. Models in which BMI was modelled as a linear term starting at BMI 20, a value which corresponds the lower limit of the reference BMI interval, were calculated (HR per 5 BMI units) as well as models in which BMI were modelled with restricted cubic splines.

Models with splines had BMI as cubic spline with four knots (5%, 35%, 65% and 95%) and were stratified by year (5-year age group) and parity, model 2 was further adjusted by diabetes and hypertension at baseline and model 3 was additionally adjusted by smoking and education. Further analyses assessed whether the association between BMI and obesity-related cancer forms were non-linear by comparing the log-likelihood (deviance) of the models with BMI as spline as a linear term. The p-value is for the test of non-linearity. The non-linearity was tested for BMI above 20. The spline analysis allows the exposure (BMI) to have any association with the outcome, including a non-linear association in the lower range of BMI.

It is possible that cancers were already present at baseline but not detected until after the inclusion. We therefore performed a sensitivity analysis where the follow-up started 1 year after inclusion.

Crude incidence rates and 95% CI were calculated using Poisson regression for each BMI group separately. We used 2-sided P values and considered a P value of < 0.05 as statistically significant. All statistical calculations were performed using R software, version 4.2.2 (http://www.R-project.org).

## Results

Among 1,393,346 potentially eligible women aged 18–45 years, 1447 were excluded due to incorrect data on height or weight, and 5174 because of previous cancer. The final study thus comprised 1,386,725 women. (Supplementary Fig. 1). The mean age among these participants was 27.9 [standard deviation (SD) 4.9] years and the median follow-up time was 16.3 [interquartile range (IQR) 7.7–23.5] years. The mean BMI at baseline (first antenatal visit) was 23.7 (SD 4.0); 69.1% had normal BMI (BMI 18.5–< 25), 20.1% had overweight (BMI 25–< 30), and 7.6% fulfilled criteria for obesity (BMI ≥ 30). Preexisting disorders were rare in this young population, with diabetes mellitus (0.59%), hypertension (0.41%) the most common. About 15% were smokers with the highest prevalence among the leanest two categories. Education longer than 12 years was more common in women of normal weight (BMI 18.5–< 25) than in those with underweight or overweight/obesity (Table 1).

**Table 1:** Baseline characteristics in women aged 18–45 years by body mass index (BMI) categories.

Number of women	All	BMI < 18.50	BMI 18.5– < 20	BMI 20– < 22.5*	BMI 22.5– < 25	BMI 25– < 30	BMI ≥ 30
	n = 1,386,725	n = 46,174	n = 140,725	n = 450,766	n = 366,078	n = 278,103	n = 104,879
% of total	100%	3.33%	10.15%	32.51%	26.40%	20.05%	7.56%
Age, years ± SD	27.9 ± 4.9	26.3 ± 4.8	27.2 ± 4.8	27.9 ± 4.8	28.1 ± 4.9	28.2 ± 5.1	28.0 ± 5.2
Height, mean ± SD	166 ± 6.3	166 ± 6.5	166 ± 6.3	167 ± 6.1	166 ± 6.3	166 ± 6.4	166 ± 6.3
Weight, mean ± SD	65.5 ± 12.0	49.0 ± 4.4	53.7 ± 4.2	59.2 ± 4.7	65.4 ± 5.3	74.4 ± 69	92.5 ± 11.8
BMI, mean ± SD	23.7 ± 4.0	17.7 ± 0.7	19.4 ± 0.4	21.3 ± 0.7	23.7 ± 0.7	26.9 ± 1.4	33.6 ± 3.5
First parity n (%)	1,087,046 (78.4)	36,108 (78.2)	110,187 (78.3)	353,919 (78.5)	287,322 (78.5)	217,022 (78.0)	82,488 (78.7)
Smoking, n (%)*	201,421 (14.5)	9311 (20.2)	23,281 (16.5)	63,446 (14.1)	48,926 (13.4)	39,324(14.1)	17,133 (16.4)
Hypertension, n (%)	5643 (0.4)	108 (0.2)	321 (0.2	1272 (0.3)	1299 (0.4)	1448 (0.5)	1195 (1.1)
Diabetes, n (%)	8179 (0.6)	87 (0.2)	337 (0.2)	1734 (0.4)	2315 (0.6)	2394 (0.9)	1312 (1.3)
Atrial fibrillation, n (%)	212 (0.1)	7 (0.1)	21 (0.1)	64 (0.1)	62 (0.1)	34 (0.1)	24 (0.1)
Chronic kidney disease, n (%)	880 (0.1)	31 (0.1)	79 (0.1)	280 (0.1)	223 (0.1)	168 (0.1)	99 (0.1)
Congenital heart disease, n (%)	102 (0.1)	2 (0.1)	10 (0.1)	25 (0.1)	23 (0.1)	28 (0.1)	14 (0.1)
Valvular disease, n (%)	209 (0.1)	8 (0.1)	28 (0.1)	69 (0.1)	55 (0.1)	33 (0.1)	16 (0.1)
Education, n (%)^†^							
≤ 9 years	182,542 (13.2)	9684 (21.0)	20,229 (14.4)	53,545 (11.9)	43,794 (12.1)	38,249 (13.8)	17,041 (16.3)
10–12 years	652,377 (47.0)	21,486 (46.5)	63,706 (45. 3)	202,415 (44.9)	169,912 (46.4)	137,727 (49.5)	57,131 (54.5)
> 12 years	536,431 (38.7)	14,298 (31.0)	55,044 (39.1)	189,886 (42.1)	148,595 (40.6)	99,079 (35.6)	29,529 (28.2)

* Missing information on smoking 2.6%, ^†^ Missing information on education 1.1%, BMI; body mass index, SD; standard deviation, *; reference group.

Table 2 shows the incidence of cancer by BMI categories. During follow-up, 10,812 obesity-related cancer cases occurred among 9808 (0.7%) women. The cancer-related mortality was highest at 12.6/100,000 person-years in women with BMI ≥ 30. The dominating cancer type was ovarian (21.3%), colon (19.9%), thyroid (16.3%) and endometrial cancer (14.3%). A total of 2085 (0.2%) women developed ovarian cancer, at a mean age of 45.9 (SD 9.1) years, increasing moderately with increasing BMI, from 8.3 to 10.7 /100,000 person-years in those with BMI < 18.5 and BMI ≥ 30, respectively. Thyroid cancer also increased with higher BMI and was slightly more common among women with obesity (8.1, compared to 6.9 per 100,000 person-years among women with low normal BMI (20–< 22.5). The leanest women had the lowest incidence of endometrial cancer, with rates essentially doubling comparing those with underweight (BMI < 18.5) with those with obesity (BMI ≥ 30), from 4.7 to 10.1 /100,000 person-years. Of the less common cancer types (≤ 3%), esophageal and gastric cancer (cardia) was considerably more common among those with BMI ≥ 30, and so was pancreatic, renal cell carcinoma, gallbladder, and liver cancer (Table 2).

**Table 2:** Incidence of obesity-related cancer forms in women aged 18–45 years by body mass index (BMI) categories.

Number of women	All	BMI < 18.50	BMI 18.5–< 20	BMI 20–< 22.5*	BMI 22.5–< 25	BMI 25–< 30	BMI ≥ 30
	n = 1 386 725	n = 46 174	n = 140 725	n = 450 766	n = 366 078	n = 278 103	n = 104 879
% of total	100%	3.33%	10.15%	32.51%	26.40%	20.05%	7.56%
Total cancer, n	9808	335	993	3341	2565	1843	730
Age at cancer diagnosis, years ± SD	46.6 ± 9.4	46.5 ± 9.8	47.1 ± 9.4	46.8 ± 9.3	46.6 ± 9.4	46.0 ± 9.5	46.4 ± 9.8
Cases per 100,000 person-years (95% CI)	44.1 (43.2–45.0)	41.6 (37.3–46.3)	40.3 (37.8–42.9)	43.3 (41.8–44.8)	43.8 (42.1–45.5)	45.5 (43.5–47.6)	54.1 (50.2–58.1)
Cancer death, n	2044	74	203	711	527	359	170
Age at death, years ± SD	49.9 ± 8.5	48.8 ± 10.2	50.6 ± 8.7	49.8 ± 8.3	50.6 ± 8.5	49.0 ± 8.3	49.9 ± 8.9
Cases per 100,000 person-years (95% CI)	9.2 (8.8–9.6)	9.2 (7.2–11.5)	8.2 (7.1–9.4)	9.2 (8.5–9.9)	9.0 (8.2–9.8)	8.8 (8.0–9.8)	12.6 (10.7–14.6)
Multiple myeloma, n	349	13	35	118	99	61	23
Age at multiple myeloma diagnosis, years ± SD	48.7 ± 8.4	51.5 ± 6.7	49.1 ± 9.6	48.0 ± 8.0	48.5 ± 8.3	48.2 ± 8.5	51.9 ± 9.8
Cases per 100,000 person-years (95% CI)	1.6 (1.4–1.7)	1.6 (0.9–2.8)	1.4 (1.0–2.0)	1.5 (1.3–1.8)	1.7 (1.4–2.1)	1.5 (1.1–1.9)	1.7 (1.1–2.6)
Endometrial cancer, n	1398	38	133	451	347	293	136
Age at endometrial cancer diagnosis, years ± SD	50.0 ± 8.4	50.3 ± 8.7	51.1 ± 8.0	50.1 ± 8.3	49.4 ± 8.7	50.0 ± 8.5	49.8 ± 8.5
Cases per 100,000 person-years (95% CI)	6.3 (5.9–6.6)	4.7 (3.3–6.5)	5.4 (4.5–6.4)	5.8 (5.3–6.4)	5.9 (5.3–6.6)	7.2 (6.4–8.1)	10.1 (8.4–11.9)
Ovarian cancer, n	2085	67	235	707	551	380	145
Age at ovarian cancer diagnosis, years ± SD	45.9 ± 9.1	45.8 ± 9.6	47.2 ± 8.9	45.9 ± 9.0	46.5 ± 9.3	44.8 ± 9.1	44.1 ± 9.2
Cases per 100,000 person-years (95% CI)	9.4 (9.0–9.8)	8.3 (6.4–10.6)	9.5 (8.3–10.8)	9.1 (8.5–9.8)	9.4 (8.6–10.2)	9.4 (8.4–10.4)	10.7 (9.0–12.6)
Esophageal cancer, n	132	5	13	44	34	22	14
Age at esophageal cancer diagnosis, years ± SD	49.6 ± 8.8	44.6 ± 11.9	52.4 ± 12.0	50.4 ± 9.1	49.2 ± 7.2	48.9 ± 8.6	48.0 ± 7.3
Cases per 100,000 person-years (95% CI)	0.6 (0.5–0.7)	0.6 (0.2–1.4)	0.5 (0.3–0.9)	0.6 (0.4–0.8)	0.6 (0.4–0.8)	0.5 (0.3–0.8)	1.0 (0.6–1.7)
Gastric cancer (cardia), n	115	3	8	49	27	15	13
Age at gastric cancer diagnosis, years ± SD	45.0 ± 9.3	33.7 ± 12.8	43.3 ± 10.2	44.5 ± 8.8	49.2 ± 9.1	42.69.7	44.4 ± 7.4
Cases per 100,000 person-years (95% CI)	0.5 (0.4–0.6)	0.4 (0.1–1.1)	0.3 (0.1–0.6)	0.6 (0.5–0.8)	0.5 (0.3–0.7)	0.4 (0.2–0.6)	1.0 (0.5–1.6)
Gallbladder cancer, n	281	8	24	98	79	45	27
Age at gallbladder cancer diagnosis, years ± SD	49.7 ± 8.5	45.9 ± 10.9	50.6 ± 8.8	49.7 ± 7.2	50.2 ± 9.2	49.3 ± 8.3	49.0 ± 10.1
Cases per 100,000 person-years (95% CI)	1.3 (1.1–1.4)	1.0 (0.4–2.0)	1.0 (0.6–1.4)	1.3 (1.0–1.5)	1.3 (1.1–1.7)	1.1 (0.8–1.5)	2.0 (1.3–2.9)
Thyroid cancer, n	1600	51	181	531	426	302	109
Age at thyroid cancer diagnosis, years ± SD	40.2 ± 8.6	39.4 ± 9.4	40.5 ± 9.2	40.6 ± 8.8	40.3 ± 8.5	39.9 ± 8.1	39.4 ± 8.6
Cases per 100,000 person-years (95% CI)	7.2 (6.8–7.5)	6.3 (4.7–8.3)	7.3 (6.3–8.5)	6.9 (6.3–7.5)	7.3 (6.6–8.0)	7.4 (6.6–8.3)	8.1 (6.6–9.7)
Pancreas cancer, n	659	24	57	207	182	142	47
Age at pancreas cancer diagnosis, years ± SD	50.0 ± 8.5	46.9 ± 8.8	51.0 ± 9.4	50.0 ± 8.2	50.7 ± 8.5	49.4 ± 8.6	49.8 ± 8.7
Cases per 100,000 person-years (95% CI)	3.0 (2.7–3.2)	3.0 (1.9–4.4)	2.3 (1.7–3.0)	2.7 (2.3–3.1)	3.1 (2.7–3.6)	3.5 (2.9–4.1)	3.5 2.6–4.6)
Liver cancer, n	343	8	38	131	85	51	30
Age at liver cancer diagnosis, years ± SD	47.5 ± 9.6	40.8 ± 9.7	49.5 ± 10.1	46.3 ± 9.7	48.5 ± 9.3	49.2 ± 9.4	46.4 ± 9.6
Cases per 100,000 person-years (95% CI)	1.5 (1.4–1.7)	1.0 (0.4–2.0)	1.5 (1.1–2.1)	1.7 (1.4–2.0)	1.4 (1.2–1.8)	1.3 (0.9–1.7)	2.2 (1.5–3.2)
Colon cancer, n	1953	82	185	716	498	345	127
Age at colon cancer diagnosis, years ± SD	48.0 ± 9.2	49.2 ± 9.2	48.3 ± 8.5	48.6 ± 8.8	47.6 ± 9.2	46.8 ± 9.8	48.5 ± 10.2
Cases per 100,000 person-years (95% CI)	8.8 (8.4–9.2)	10.2 (8.1–12.6)	7.5 (6.4–8.7)	9.3 (8.6–10.0)	8.5 (7.8–9.3)	8.5 (7.6–9.4)	9.4 (7.8–11.2)
Rectal cancer, n	1335	48	138	470	374	224	81
Age at rectal cancer diagnosis, years ± SD	48.8 ± 8.1	50.0 ± 7.6	49.4 ± 6.5	49.6 ± 8.2	48.1 ± 8.2	47.8 ± 8.4	48.5 ± 9.2
Cases per 100,000 person-years (95% CI)	6.0 (5.7–6.3)	6.0 (4.4–7.9)	5.6 (4.7–6.6)	6.1 (5.6–6.7)	6.4 (5.7–7.0)	5.5 (4.8–6.3)	6.0 (4.8–7.4)
Renal cell carcinoma, n	562	166	21	51	137	134	53
Age at renal cell carcinoma diagnosis, years ± SD	47.3 ± 894	47.4 ± 8.67	48.2 ± 9.55	46.7 ± 9.41	47.5 ± 9.46	47.3 ± 8.46)	47.3 ± 9.27
Cases per 100,000 person-years (95% CI)	2.5 (2.3–2.7)	2.1 (1.8–2.5)	2.6 (1.6–4.0)	2.1 (1.5–2.7)	2.3 (2.0–2.8)	3.3 (2.8–3.9)	3.9 (2.9–5.1)

Data presented as mean ± SD except for event count (n) and event rate [events/100,000 years (95% CI)]. *CI* confidence interval, *IQR* interquartile range, *SD* standard deviation, *reference group.

**Figure 1:**
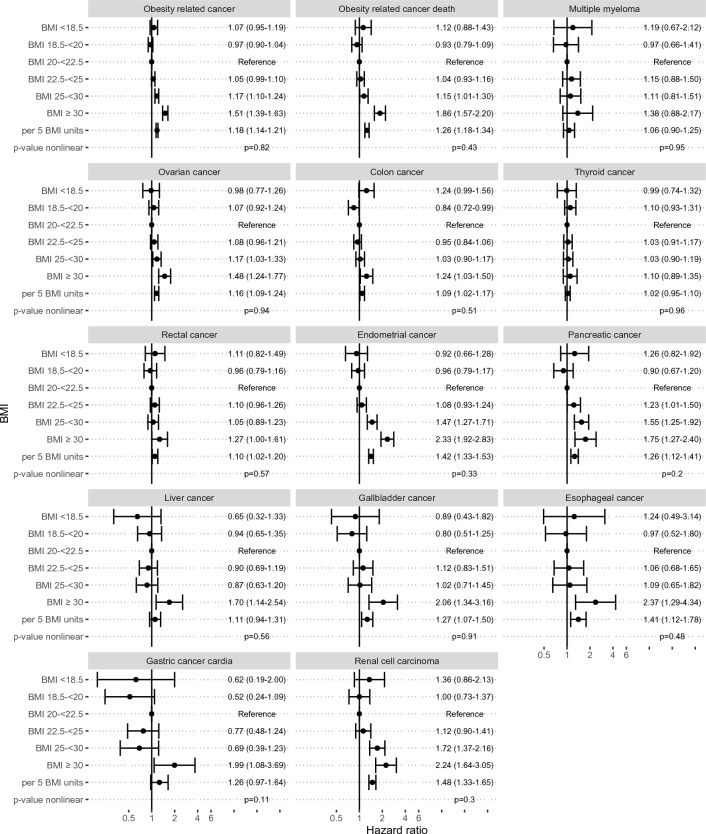
Risk of obesity-related cancer forms in women aged 18–45 years by body mass index (BMI) categories, expressed as hazard ratios with 95% confidence intervals. Hazard ratios for linear increase in BMI was calculated for a 5-unit BMI increase for BMI above 20, test for nonlinearity was performed by comparing the log-likelihood of models with BMI as a linear term and as a spline.

In the calculation of HRs of overall cancer, cancer deaths, and individual cancer types, BMI 20–< 22.5 was used as reference category. There was a weak association between BMI and risk of all obesity-related cancer types analyzed together (Fig. 1). For cancer death, there was an increased risk in women with obesity (BMI ≥ 30); adjusted HR of 1.86 (95% CI 1.57–2.20 compared to women within the reference BMI category. The highest HR for colon cancer was observed in women with obesity (BMI ≥ 30), with HR 1.24 (95% CI 1.03–1.50), while no other BMI group displayed any increased risk (Fig. 1).

The highest statistically significant risks for renal cell carcinoma and gallbladder cancer were found in women with BMI ≥ 30 (adjusted HR 2.24, 95% CI 1.64–3.05; and 2.06, 95% CI 1.34–3.16, respectively (Fig. 1). The adjusted HRs for endometrial cancer increased markedly with increasing BMI and was 1.47 (95% CI 1.27–1.71) in women with overweight and 2.33 (95% CI 1.92–2.83) in those with obesity. The adjusted HR for ovarian cancer increased gradually with higher BMI to HRs of 1.17 (95% CI 1.03–1.33) and 1.48 (95% CI 1.24–1.77) for women with overweight and obesity, respectively. No increased risk of thyroid cancer was observed in relation to BMI (Fig. 1). Linear and positive associations were found between BMI and incident cancer in the ovary, colon, endometrium, pancreas, gallbladder, esophagus, rectal cancer, renal cell carcinoma and any obesity related cancer, as well as death from obesity-related cancer forms (Supplementary Table 3). The adjusted HR of these cancers increased with higher BMI when we also adjusted for diabetes, hypertension at baseline, in addition to prior adjustments (age, year of pregnancy and parity status) (Supplementary Table 4).

Possibly, some cancers could have been present already at index, but only detected after the index date. In total, 1.6% of the subjects had a follow up shorter than one year and within this period < 1.5% of the total cancer events occurred. Excluding these early cases did not affect any results (Supplementary Fig. 2).

The incidence rates of cancer according to organ system are shown in Supplementary Table 5, which includes cancers not associated with BMI. Overall, 21,101 women (1.5%) were diagnosed with breast cancer, at a mean age of 46.8 (SD 7.7) years, with the highest rates in the underweight women (BMI < 18.5) and normal BMI categories (BMI 18.5–< 25) with rates per 100,000 person-years of 87.4 to 104.0 After breast cancer, the majority were skin cancer and melanoma with the highest incidence rates of 101.4 and 99.8/100,000 person-years found in women with BMI 18.5–< 20 and 20–< 22.5, respectively, and an inverse association with BMI. Cancer in female genital organs had the highest incidence of 35.1/100,000 person-years in women with BMI ≥ 30. The highest incidence rates of cancer in respiratory organs were observed among underweight and lean women (12.0 and 9.5/100,000 person-years).

Women with BMI ≥ 30 had the highest risk of developing cancer in digestive organs of 24.9/100,000 person-years, compared to women with normal weight. Cancer in urinary tract tended to be more prevalent in women with overweight and obesity, with the highest rates in women with BMI ≥ 30, at 6.9/100,000 person-years, compared to low-normal weight women. There was no association between BMI and cancer in the bone or articular organs (Supplementary Table 5).

**Figure 2:**
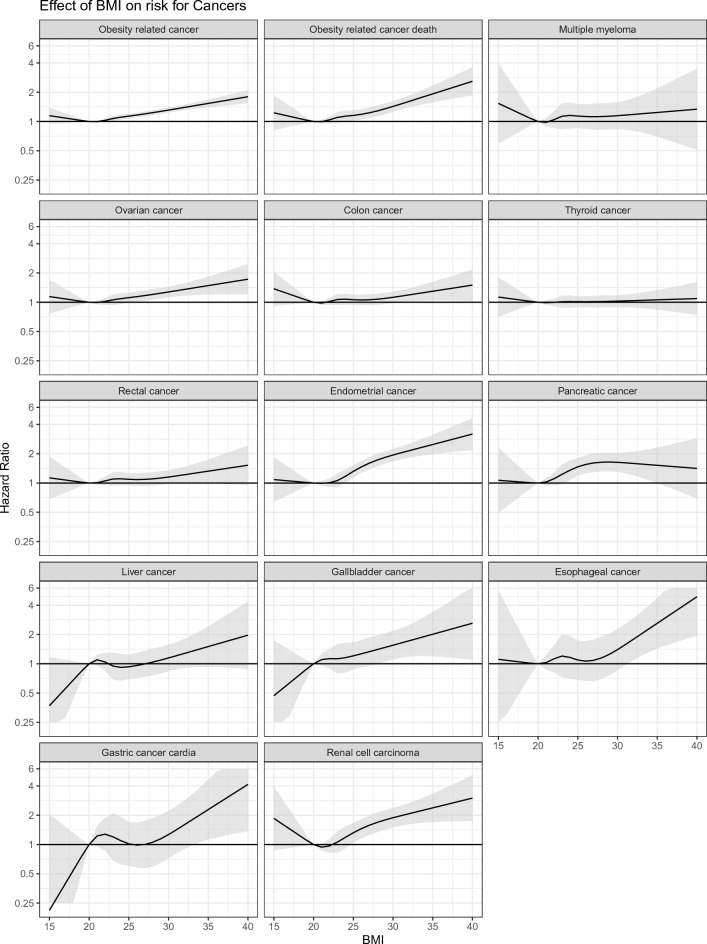
Spline model on the effect of body mass index on the risk for obesity-related cancer incidence and mortality, and breast cancer (Model 3 adjusted for year of pregnancy, parity, diabetes, hypertension and further for smoking and education at baseline). The spline analysis allows the BMI to have any association with the outcome over the full range of BMI, including a non-linear association in the lower range.

Supplementary Fig. 3 shows the HRs of all cancer types according to organ system. There were inverse associations between BMI and any cancer, breast cancer, and skin cancer. The highest risk for cancer in the genital organs was found in women with BMI ≥ 30 (adjusted HR 1.34, 95% CI 1.21–1.48). The highest risk for cancer of the digestive organs and urinary tract was found in women with BMI ≥ 30 (HR 1.48, 95% CI 1.31–1.67; and 1.96, 95% CI 1.56–2.47, respectively). The risk for cancer of hematopoietic tissue was increased in women with BMI ≥ 30 (HR 1.41, 95% CI 1.22–1.64). There was no statistically significant association between BMI and risk of malignant neoplasms of endocrine glands or the central nervous system. Positive and linear associations were found between BMI and endometrial cancer, ovarian cancer, rectal cancer, gallbladder cancer, and esophageal cancer (Fig. 2, Supplementary Figs. 4–5).

## Discussion

This cohort study of women of childbearing age at baseline revealed significant heterogeneity in the risk of cancer across BMI categories. Higher, compared to reference BMI levels, were associated with increased risks of endometrial and ovarian cancer. Low BMI was associated with cancers not regarded as obesity-related such as malignancies in respiratory system, which are related to smoking. The highest HR for mortality from any type of cancer was found in women with BMI ≥ 30. Linear and positive associations were found between BMI and incident cancer in the ovary, colon, endometrium, pancreas, gallbladder, kidney, esophagus, and rectum, as well as death from obesity-related cancer forms.

In a cross-sectional US population-based study there was an almost 30% increase over time in the incidence rate of many cancer types in adolescents and young adults from 1973 to 2015, particularly kidney, thyroid, and colorectal cancer^2^. The increase in obesity-related cancer types may well be due to increasing rates of obesity, and to changes in diagnostic screening. Incidence of other cancer types that are not obesity related, such as lung cancer decreased over time^2^, likely due to lower smoking rates.

Breast cancer is the most common cancer in women^5^. where previous studies have found that obesity is associated with postmenopausal breast cancer^4,19,20^, but with an inverse association between high BMI and premenopausal breast cancer, consistent with previous studies^20,21^. The women in our study, aged 18–45 years and on average younger than 50 years at diagnosis, were probably largely premenopausal. Epidemiological studies have found a positive association between breast cancer and high levels of endogenous estrogen and progesterone in postmenopausal women^22^. Postmenopausal women with obesity have increased insulin resistance and increased conversion of androgens to estrogens in adipose tissue that could influence the development of breast cancer through increased cell proliferation and decreased apoptosis^22^. Obesity is also associated with increased secretion of cytokines in adipose tissue which promote cancer cell growth^23^. Early life and midlife obesity have been shown to have opposite effects on postmenopausal breast cancer risk and the biological mechanisms underlying these associations are likely to differ^24,25^.

Similar to the Cancer Prevention study II Nutrition cohort, our study found that high BMI at age 18–45 years was associated with increased risk for endometrial cancer, but unlike that study we had no data on adult weight gain which was also found to be associated with increased risk^26^. Obesity is one of the major risk factors for type 2 diabetes that in turn predisposes to endometrial cancer^27^. Women with diabetes, a largely obesity-related condition, have an up to 72% increased risk of endometrial cancer, compared to those without diabetes^28^. Obesity is associated with elevated levels of estrogens due to hormonal conversion (androstenedione to estrone) in adipose tissue that may lead to endometrial proliferation and which in some cases could progress into endometrial cancer^29^.

While the association between BMI and endometrial cancer has long been established^10^, the association between obesity and risk of ovarian cancer has been more in doubt. In a Norwegian study that included almost 1 million women, those with a high BMI in adolescence or young adulthood had an increased risk of ovarian cancer^30^. We also found a positive association between higher BMI categories and risk of ovarian comparing BMI ≥ 30 to the reference category of BMI 20–< 22.5. A meta-analysis of 30 studies reported that for a 5-unit increase in BMI, the risk of colon cancer increased in both men (RR 1.30, 95% CI 1.25–1.35) and women (RR 1.12, 95% CI 1.07–1.18) but this association was more consistent in men^31^. We found a weak linear association between BMI and colon cancer, where women with BMI ≥ 30 had the highest risk of colon cancer compared to the reference group. Notably, our estimate for the linear association was numerically similar to that in the women of that study (HR 1.13, 95% CI 1.03–1.24).

In a recent comprehensive review, most of the studies included were observational studies on cancer risk and obesity, chiefly centered around adult BMI. In general, positive dose–response relationships were found in our study for cancers of the ovary, colon, gastric cardia, liver, gallbladder, pancreas, and kidney and for esophageal adenocarcinoma, with risk estimates consistent with the collective data from this review^8^.

### Strengths and limitations

Among strengths of our study is the inclusion of almost all women who gave birth in Sweden during the study period, representing a large majority of women (almost 85% of women in Sweden), and the long and complete follow-up. The study used registry-based data from a nationwide cohort that includes almost all (99%) women who gave birth in Sweden, with universal health coverage. There are also limitations. The study was based on routinely collected administrative data, hence, information on other potential risk factors such as genetic factors, alcohol intake or dietary habits, were not available. There is a possible delay in cancer detection and it is possible that cancers were already present at baseline. Sensitivity analysis, excluding the first year of follow up did not show any difference in the results. Data on weight was not standardized, and in some instances only recorded as 2 digits (up to 99 kg) and in these instances calculated from weight at delivery minus weight gain during pregnancy. However, in sensitivity analyses that we performed, excluding these subjects resulted in a biased sample and too few cases to provide meaningful information. Another limitation was the lack of information on changes in BMI during the follow-up. However, high BMI levels are likely to persist into middle age. Furthermore, we did not have data of menopausal status, however, the mean age of diagnosis for breast cancer was 46.8 years which puts most of the women afflicted at an age when they were likely premenopausal. Also, there were no data for other measurements of obesity other than BMI and we had no information on some cancer subtypes, e.g. breast cancer by hormone receptor status. Finally, as only women who giving birth were included in the study, results may not be generalizable to all women.

## Conclusions

In conclusion, this large, population-based study found that higher BMI in young women was associated with an increased risk of most obesity-related cancer types, evident already at high normal levels of BMI.

### Supplementary Information

Supplementary Information.

## Data Availability

The data that support the findings of our study are available from the Swedish National Board of Health and Welfare and Statistics Sweden. For legal reasons, these datasets are not directly available from the corresponding author. However, researchers can apply for these data by contacting these government agencies, fulfilling legal and regulatory requirements, and providing an acceptance letter from the Swedish Ethical Review Authority.

## References

[1] Dagenais GR (2020). Variations in common diseases, hospital admissions, and deaths in middle-aged adults in 21 countries from five continents (PURE): A prospective cohort study. Lancet.

[2] Scott AR (2020). Trends in cancer incidence in US adolescents and young adults, 1973–2015. JAMA Netw. Open.

[3] Stein C, Colditz G (2004). Modifiable risk factors for cancer. Br. J. Cancer.

[4] Bhaskaran K (2014). Body-mass index and risk of 22 specific cancers: A population-based cohort study of 5.24 million UK adults. Lancet.

[5] (Socialstyrelsen), T.N.B.o.H.a.W. *Statistics of Causes of Death by gender 2018.* (2019).

[6] Dikaiou P (2020). Obesity, overweight and risk for cardiovascular disease and mortality in young women. Eur. J. Prev. Cardiol..

[7] Hossain P, Kawar B, El Nahas M (2007). Obesity and diabetes in the developing world: A growing challenge. N. Engl. J. Med..

[8] Lauby-Secretan B (2016). Body fatness and cancer-viewpoint of the IARC working group. N. Engl. J. Med..

[9] Renehan AG (2008). Body-mass index and incidence of cancer: A systematic review and meta-analysis of prospective observational studies. Lancet.

[10] Bjorge T (2019). BMI and weight changes and risk of obesity-related cancers: A pooled European cohort study. Int. J. Epidemiol..

[11] Furer A (2020). Adolescent obesity and midlife cancer risk: A population-based cohort study of 2.3 million adolescents in Israel. Lancet Diabetes Endocrinol..

[12] Birks S (2012). A systematic review of the impact of weight loss on cancer incidence and mortality. Obes. Rev..

[13] Collaboration NRF (2016). Trends in adult body-mass index in 200 countries from 1975 to 2014: A pooled analysis of 1698 population-based measurement studies with 19.2 million participants. Lancet.

[14] Chaparro MP (2015). Regional inequalities in pre-pregnancy overweight and obesity in Sweden, 1992, 2000, and 2010. Scand. J. Public Health.

[15] (Socialstyrelsen), T.N.B.o.H.a.W. *The Swedish Medical Birth Register: A Summary of Content and Quality.* (2003).

[16] Cnattingius S (2023). The Swedish medical birth register during five decades: Documentation of the content and quality of the register. Eur. J. Epidemiol..

[17] Persson CE (2018). Young women, body size and risk of atrial fibrillation. Eur. J. Prev. Cardiol..

[18] Grambsch PM, Therneau TM (1994). Proportional hazards tests and diagnostics based on weighted residuals. Biometrika.

[19] Reeves GK (2007). Cancer incidence and mortality in relation to body mass index in the Million Women Study: Cohort study. BMJ.

[20] Schoemaker MJ (2018). Association of body mass index and age with subsequent breast cancer risk in premenopausal women. JAMA Oncol..

[21] Urbute A, Frederiksen K, Kjaer SK (2022). Early adulthood overweight and obesity and risk of premenopausal ovarian cancer, and premenopausal breast cancer including receptor status: Prospective cohort study of nearly 500,000 Danish women. Ann. Epidemiol..

[22] Zhang X (2015). Adult body size and physical activity in relation to risk of breast cancer according to tumor androgen receptor status. Cancer Epidemiol. Prev. Biomark..

[23] Hopkins BD, Goncalves MD, Cantley LC (2016). Obesity and cancer mechanisms: Cancer metabolism. J. Clin. Oncol..

[24] Missmer SA (2004). Endogenous estrogen, androgen, and progesterone concentrations and breast cancer risk among postmenopausal women. J. Natl. Cancer Inst..

[25] Yang TO (2022). Body size in early life and the risk of postmenopausal breast cancer. BMC Cancer.

[26] Stevens VL (2014). Body weight in early adulthood, adult weight gain, and risk of endometrial cancer in women not using postmenopausal hormones. Cancer Causes Control.

[27] Aune D (2015). Anthropometric factors and endometrial cancer risk: A systematic review and dose–response meta-analysis of prospective studies. Ann. Oncol..

[28] Saed L (2019). The effect of diabetes on the risk of endometrial Cancer: An updated a systematic review and meta-analysis. BMC Cancer.

[29] Mauras N (2015). Estrogens and their genotoxic metabolites are increased in obese prepubertal girls. J. Clin. Endocrinol. Metab..

[30] Engeland A, Tretli S, Bjørge T (2003). Height, body mass index, and ovarian cancer: A follow-up of 1.1 million Norwegian women. J. Natl. Cancer Inst..

[31] Larsson SC, Wolk A (2007). Obesity and colon and rectal cancer risk: A meta-analysis of prospective studies. Am. J. Clin. Nutr..

