# Supplementary material for: Body mass index and risk of cancer in young women
Source: Sci Rep. 2024 Mar 15;14:6245. doi: 10.1038/s41598-024-56899-1 (PMC10940279; doi:10.1038/s41598-024-56899-1)

**Supplementary Material**

**Supplementary** **Table 1**. Obesity-related cancer diagnosis codes according to the International Statistical Classification of Diseases and Related Health Problems (eighth, ninth and tenth revision).

**Supplementary** **Table 2**. Diagnosis codes of cancer by organ system according to the International Statistical Classification of Diseases and Related Health Problems (eighth, ninth and tenth revision).

**Supplementary** **Table 3.** Hazard ratio (HR) and 95% confidence intervals (CI) for obesity-related cancer forms in relation to body mass index (BMI) in young women, adjusted for year (5-year age group) of pregnancy and parity at baseline (Model 1).

**Supplementary Table 4.** Hazard ratio (HR) and 95% confidence intervals (CI) for obesity-related cancer forms in relation to body mass index (BMI) in young women, adjusted for year (5-year age group) year of pregnancy, parity, diabetes and hypertension at baseline (Model 2).

**Supplementary Table 5.** Incidence of cancer forms by organ system in women aged 18-45 years by body mass index (BMI) categories.

**Supplementary** **Figure 1**. Flowchart of the study population.

**Supplementary Figure 2**. Hazard ratios of cancer forms in women aged 18-45 years by body mass index categories, exclude individuals with diagnosis of cancer the first year after the registration.

**Supplementary Figure** **3**. Risk of cancer by organ system in women aged 18-45 years by body mass index (BMI) categories, expressed as hazard ratios with 95% confidence intervals. Hazard ratios for linear increase in BMI was calculated for a 5-unit BMI increase for BMI above 20, test for nonlinearity was performed by comparing the log-likelihood of models with BMI as a linear term and as a spline.

**Supplementary Figure 4**. Spline model on the effect of body mass index on the risk for obesity-related cancer incidence and mortality, and breast cancer (Model 1 adjusted for year of pregnancy and parity).

**Supplementary Figure 5**. Spline model on the effect of body mass index on the risk for obesity-related cancer incidence and mortality, and breast cancer (Model 2 adjusted for year of pregnancy, parity, and for diabetes and hypertension at baseline).

**Supplementary** **Table 1**. Obesity-related cancer diagnosis codes according to the International Statistical Classification of Diseases and Related Health Problems (eighth, ninth and tenth revision)

| **Obesity-related cancer** | **ICD 8** | **ICD 9*** | **ICD 10** |
| --- | --- | --- | --- |
| Esophageal cancer | 150 | 150 | C15 |
| Gastric cancer (cardia) | 151.0 | 151.0 | C16.0 |
| Colon cancer | 153 | 153 | C18 |
| Rectal cancer | 154 | 154 | C19-21 |
| Liver cancer | 155 | 155 | C22 |
| Gallbladder cancer | 156 | 156 | C23-24 |
| Pancreas cancer | 157 | 157 | C25 |
| Multiple myeloma | 203 | 203 | C90 |
| Endometrial cancer | 182 | 182 | C54 |
| Ovarian cancer | 183 | 183 | C56 |
| Renal cell carcinoma | 189.0 | 189A | C64 |
| Thyroid cancer | 193 | 193 | C73 |

ICD, International Classification of Diseases. *Swedish version of the ICD-9.

**Supplementary** **Table 2**. Diagnosis codes of cancer by organ system according to the International Statistical Classification of Diseases and Related Health Problems (eighth, ninth and tenth revision).

| **Diagnosis** | **ICD-8** | **ICD-9*** | **ICD-10** |
| --- | --- | --- | --- |
| Malignant neoplasms of lip, oral cavity and pharynx (1) | 140-149 | 140-149 | C00-C14 |
| Malignant neoplasms of digestive organs (2) | 150-159 | 150-159 | C15-C26 |
| Malignant neoplasms of respiratory and intrathoracic organs (3) | 160-163 | 160-165 | C30-C39 |
| Malignant neoplasms of bone and articular cartilage (4) | 170-171 | 170 | C40-C41 |
| Melanoma and other malignant neoplasms of skin (5) | 172-173 | 172-173 | C43-C44 |
| Malignant neoplasms of mesothelial and soft tissue (6) | - | 158,162,163,171,173 | C45-C49 |
| Malignant neoplasms of breast (7) | 174 | 174-175 | C50 |
| Malignant neoplasms of female genital organs (8) | 180-184 | 179-184,236C | C51-C58 |
| Malignant neoplasms of urinary tract (9) | 188-189 | 188-189 | C64-C68 |
| Malignant neoplasms of eye, brain and  other parts of central nervous system (10) | 190-192 | 190-192 | C69-C72 |
| Malignant neoplasms of thyroid  and other endocrine glands (11) | 193-194 | 193-194 | C73-C75 |
| Malignant neoplasms, stated or presumed to be primary,  of lymphoid, haematopoietic and related tissue (12) | 200-209 | 200-209 | C81-C96 |

**Supplementary Table 3.** Hazard ratio (HR) and 95% confidence intervals (CI) for obesity-related cancer forms in relation to body mass index (BMI) in young women, adjusted for year (5-year age group) and parity at baseline (Model 1).

|  | **BMI <18.5** | **BMI 18.5-<20** | **BMI 20-<22.5** | **BMI 22.5-<25** | **BMI 25-<30** | **BMI ≥ 30** | **HR per 5 BMI units** | **P value** |
| --- | --- | --- | --- | --- | --- | --- | --- | --- |
| **Colon_cancer, n** | 82 | 185 | 716 | 498 | 345 | 127 | 970 |  |
| HR (95 % CI) | 1.24 (0.99-1.56) | 0.84 (0.72-0.99) | Reference | 0.95 (0.84-1.06) | 1.03 (0.90-1.17) | 1.24 (1.03-1.50) | 1.09 (1.02-1.17) | 0.51 |
| **Endometrial cancer, n** | 38 | 133 | 451 | 347 | 293 | 136 | 776 |  |
| HR (95 % CI) | 0.92 (0.66-1.28) | 0.96 (0.79-1.17) | Reference | 1.08 (0.93-1.24) | 1.47 (1.27-1.71) | 2.33 (1.92-2.83) | 1.42 (1.33-1.53) | 0.33 |
| **Gallbladder cancer, n** | 8 | 24 | 98 | 79 | 45 | 27 | 151 |  |
| HR (95 % CI) | 0.89 (0.43-1.82) | 0.80 (0.51-1.25) | Reference | 1.12 (0.83-1.51) | 1.02 (0.72-1.45) | 2.06 (1.34-3.16) | 1.27 (1.07-1.50) | 0.91 |
| **Gastric cancer cardia, n** | 3 | 8 | 49 | 27 | 15 | 13 | 55 |  |
| HR (95 % CI) | 0.62 (0.19-1.99) | 0.52 (0.24-1.09) | Reference | 0.77 (0.48-1.24) | 0.69 (0.39-1.23) | 1.99 (1.08-3.69) | 1.26 (0.97-1.64) | 0.11 |
| **Liver cancer, n** | 8 | 38 | 131 | 85 | 51 | 30 | 166 |  |
| HR (95 % CI) | 0.65 (0.32-1.32) | 0.94 (0.65-1.35) | Reference | 0.90 (0.69-1.19) | 0.87 (0.63-1.20) | 1.70 (1.14-2.54) | 1.11 (0.94-1.31) | 0.56 |
| **Obesity related cancer, n** | 335 | 993 | 3341 | 2566 | 1843 | 730 | 5 139 |  |
| HR (95 % CI) | 1.07 (0.95-1.19) | 0.97 (0.90-1.04) | Reference | 1.05 (0.99-1.10) | 1.17 (1.10-1.24) | 1.51 (1.39-1.63) | 1.18 (1.14-1.21) | 0.82 |
| **Obesity related cancer Death, n** | 74 | 203 | 711 | 527 | 359 | 170 | 1 056 |  |
| HR (95 % CI) | 1.12 (0.88-1.43) | 0.93 (0.79-1.09) | Reference | 1.04 (0.93-1.16) | 1.15 (1.01-1.30) | 1.86 (1.57-2.20) | 1.26 (1.18-1.34) | 0.43 |
| **Esophageal cancer, n** | 5 | 13 | 44 | 34 | 22 | 14 | 70 |  |
| HR (95 % CI) | 1.24 (0.49-3.14) | 0.97 (0.52-1.80) | Reference | 1.06 (0.68-1.65) | 1.09 (0.65-1.82) | 2.37 (1.29-4.34) | 1.41 (1.12-1.78) | 0.48 |
| **Ovarian cancer, n** | 67 | 235 | 707 | 551 | 380 | 145 | 1076 |  |
| HR (95 % CI) | 0.98 (0.77-1.26) | 1.07 (0.92-1.24) | Reference | 1.08 (0.96-1.21) | 1.17 (1.03-1.33) | 1.48 (1.24-1.77) | 1.16 (1.09-1.24) | 0.94 |
| **Pancreas cancer, n** | 24 | 57 | 207 | 182 | 142 | 47 | 371 |  |
| HR (95 % CI) | 1.26 (0.82-1.92) | 0.90 (0.67-1.20) | Reference | 1.23 (1.01-1.50) | 1.55 (1.25-1.92) | 1.75 (1.27-2.40) | 1.26 (1.12-1.41) | 0.20 |
| **Rectal cancer, n** | 48 | 138 | 470 | 374 | 224 | 81 | 679 |  |
| HR (95 % CI) | 1.11 (0.82-1.49) | 0.96 (0.79-1.16) | Reference | 1.10 (0.96-1.26) | 1.05 (0.89-1.23) | 1.27 (1.00-1.61) | 1.10 (1.02-1.20) | 0.57 |
| **Thyroid cancer, n** | 51 | 181 | 531 | 426 | 302 | 109 | 837 |  |
| HR (95 % CI) | 0.99 (0.74-1.32) | 1.10 (0.93-1.31) | Reference | 1.03 (0.91-1.17) | 1.03 (0.90-1.19) | 1.10 (0.89-1.35) | 1.02 (0.95-1.10) | 0.96 |
| **Multiple myeloma, n** | 13 | 35 | 118 | 99 | 61 | 23 | 183 |  |
| HR (95 % CI) | 1.19 (0.67-2.12) | 0.97 (0.66-1.41) | Reference | 1.15 (0.88-1.50) | 1.11 (0.81-1.51) | 1.38 (0.88-2.17) | 1.06 (0.90-1.25) | 0.95 |
| **Renal cell carcinoma, n** | 21 | 51 | 166 | 137 | 134 | 53 | 324 |  |
| HR (95 % CI) | 1.36 (0.86-2.13) | 1.00 (0.73-1.37) | Reference | 1.12 (0.90-1.41) | 1.72 (1.37-2.16) | 2.24 (1.64-3.05) | 1.48 (1.33-1.65) | 0.30 |

**Supplementary Table 4.** Hazard ratio (HR) and 95% confidence intervals (CI) for obesity-related cancer forms in relation to body mass index (BMI) in young women, adjusted for year (5-year age group), parity diabetes and hypertension at baseline (Model 2).

|  | **BMI <18.5** | **BMI 18.5-<20** | **BMI 20-<22.5** | **BMI 22.5-<25** | **BMI 25-<30** | **BMI ≥ 30** | **HR per 5 BMI units** | | **P value** |
| --- | --- | --- | --- | --- | --- | --- | --- | --- | --- |
| **Colon_cancer,** | 82 | 185 | 716 | 498 | 345 | 127 | 970 |  | |
| HR (95 % CI) | 1.24 (0.98-1.56) | 0.85 (0.72-0.99) | Reference | 0.95 (0.84-1.06) | 1.02 (0.90-1.16) | 1.23 (1.01-1.48) | 1.08 (1.01-1.16) | 0.51 | |
| **Endometrial cancer, n** | 38 | 133 | 451 | 347 | 293 | 136 | 776 |  | |
| HR (95 % CI) | 0.92 (0.66-1.28) | 0.96 (0.79-1.17) | Reference | 1.08 (0.93-1.24) | 1.47 (1.27-1.71) | 2.35 (1.93-2.85) | 1.43 (1.33-1.53) | 0.33 | |
| **Gallbladder cancer, n** | 8 | 24 | 98 | 79 | 45 | 27 | 151 |  | |
| HR (95 % CI) | 0.87 (0.43-1.82) | 0.80 (0.51-1.25) | Reference | 1.12 (0.83-1.50) | 1.02 (0.72-1.45) | 2.06 (1.34-3.16) | 1.27 (1.07-1.50) | 0.91 | |
| **Gastric cancer cardia, n** | 3 | 8 | 49 | 27 | 15 | 13 | 55 |  | |
| HR (95 % CI) | 0.62 (0.19-2.00) | 0.52 (0.25-1.09) | Reference | 0.77 (0.48-1.23) | 0.68 (0.38-1.22) | 1.94 (1.04-3.60) | 1.25 (0.96-1.62) | 0.11 | |
| **Liver cancer, n** | 8 | 38 | 131 | 85 | 51 | 30 | 166 |  | |
| HR (95 % CI) | 0.65 (0.312-1.33) | 0.94 (0.66-1.35) | Reference | 0.90 (0.69-1.19) | 0.86 (0.62-1.19) | 1.66 (1.11-2.48) | 1.09 (0.93-1.29) | 0.58 | |
| **Obesity related cancer, n** | 335 | 993 | 3341 | 2566 | 1843 | 730 | 5 139 | |  |
| HR (95 % CI) | 1.07 (0.95-1.20) | 0.97 (0.90-1.04) | Reference | 1.05 (0.99-1.10) | 1.17 (1.10-1.23) | 1.50 (1.38-1.62) | 1.17 (1.14-1.21) | | 0.82 |
| **Obesity related cancer Death, n** | 74 | 203 | 711 | 527 | 359 | 170 | 1 056 | |  |
| HR (95 % CI) | 1.13 (0.89-1.43) | 0.93 (0.80-1.09) | Reference | 1.04 (0.92-1.16) | 1.14 (1.00-1.30) | 1.83 (1.55-2.17) | 1.25 (1.17-1.33) | | 0.43 |
| **Esophageal cancer, n** | 5 | 13 | 44 | 34 | 22 | 14 | 70 | |  |
| HR (95 % CI) | 1.25 (0.50-3.15) | 0.97 (0.52-1.80) | Reference | 1.05 (0.67-1.64) | 1.07 (0.64-1.79) | 2.25 (1.22-4.14) | 1.38 (1.09-1.74) | | 0.49 |
| **Ovarian cancer, n** | 67 | 235 | 707 | 551 | 380 | 145 | 1 076 | |  |
| HR (95 % CI) | 0.98 (0.77-1.26) | 1.07 (0.92-1.23) | Reference | 1.08 (0.96-1.21) | 1.17 (1.04-1.33) | 1.48 (1.24-1.78) | 1.16 (1.09-1.24) | | 0.94 |
| **Pancreas cancer, n** | 24 | 57 | 207 | 182 | 142 | 47 | 371 | |  |
| HR (95 % CI) | 1.26 (0.83-1.92) | 0.90 (0.67-1.21) | Reference | 1.22 (1.00-1.49) | 1.54 (1.24-1.91) | 1.71 (1.25-2.36) | 1.25 (1.11-1.39) | | 0.19 |
| **Rectal cancer, n** | 48 | 138 | 470 | 374 | 224 | 81 | 679 | |  |
| HR (95 % CI) | 1.11 (0.82-1.49) | 0.96 (0.79-1.16) | Reference | 1.10 (0.96-1.26) | 1.05 (0.89-1.23) | 1.26 (1.00-1.60) | 1.10 (1.01-1.20) | | 0.57 |
| **Thyroid cancer, n** | 51 | 181 | 531 | 426 | 302 | 109 | 837 | |  |
| HR (95 % CI) | 0.99 (0.75-1.32) | 1.10 (0.93-1.31) | Reference | 1.03 (0.90-1.17) | 1.03 (0.90-1.19) | 1.10 (0.89-1.35) | 1.02 (0.95-1.10) | | 0.95 |
| **Multiple myeloma, n** | 13 | 35 | 118 | 99 | 61 | 23 | 183 | |  |
| HR (95 % CI) | 1.19 (0.67-2.12) | 0.97 (0.66-1.41) | Reference | 1.15 (0.88-1.50) | 1.11 (0.81-1.51) | 1.38 (0.88-2.17) | 1.06 (0.90-1.25) | | 0.95 |
| **Renal cell carcinoma, n** | 21 | 51 | 166 | 137 | 134 | 53 | 324 | |  |
| HR (95 % CI) | 1.36 (0.86-2.14) | 1.00 (0.73-1.37) | Reference | 1.12 (0.90-1.41) | 1.71 (1.36-2.15) | 2.19 (1.61-3.00) | 1.48 (1.33-1.64) | | 0.30 |

**Supplementary Table 5.** Incidence of cancer forms by organ system in women aged 18-45 years by body mass index (BMI) categories.

|  | **All** | **BMI <18.50** | **BMI 18.5–<20** | **BMI 20<22.5** | **BMI 22.5–<25** | **BMI 25–<30** | **BMI ≥30** |
| --- | --- | --- | --- | --- | --- | --- | --- |
|  |  |  |  |  |  |  |  |
| **Number of women** | **n=1 386 725** | **n=46 174** | **n=140 725** | **n=450 766** | **n=366 078** | **n=278 103** | **n=104 879** |
| **% of total** | **100%** | **3.33%** | **10.15%** | **32.51%** | **26.40%** | **20.05%** | **7.56%** |
| **Total cancer, n** | **59 186** | **2 037** | **6 804** | **21 837** | **15 616** | **9 914** | **2 978** |
| Age at diagnosis, years ± SD | 46.0±8.7 | 46.3±8.7 | 46.4±8.6 | 46.4±8.7 | 45.9±8.7 | 45.0±8.8 | 44.8±9.2 |
| Cases per 100,000 person-years (95% CI) | 269.7  (267.6-271.9) | 256.4  (254.4-267.8) | 280.2  (273.6-286.9) | 287.3  (283.5-291.1) | 270.2  (266.0-274.5) | 247.7  (242.9-252.7) | 222.7  (214.8-230.9) |
| **Cancer death, n** | **7 391** | **273** | **778** | **2 512** | **2 021** | **1 341** | **466** |
| Age at diagnosis, years ± SD | 48.0±8.8 | 48.0±9.0 | 48.7±8.5 | 48.2±8.8 | 48.2±8.7 | 47.2±8.7 | 47.8±9.1 |
| Cases per 100,000 person-years (95% CI) | 33.1  (32.4-33.9) | 33.9  (30.0-38.1) | 31.5  (29.3-33.8) | 32.5  (31.2-33.8) | 34.4  (32.9-35.9) | 33.0  (31.3-34.9) | 34.4  (31.4-37.7) |
| **Respiratory and intrathoracic organs, n** | **1915** | **97** | **234** | **665** | **486** | **332** | **101** |
| Age at diagnosis, years ± SD | 48.8±9.2 | 49.6±9.0 | 49.1±8.7 | 49.3±9.3 | 49.0±9.3 | 48.0±9.1 | 45.8±9.5 |
| Cases per 100,000 person-years (95% CI) | 8.6  (8.2-9.0) | 12.0  (9.8-14.7) | 9.5  (8.3-10.8) | 8.6  (8.0-9.3) | 8.3  (7.6-9.0) | 8.2  (7.3-9.1) | 7.5  (6.1-9.1) |
| **Breast cancer, n** | **21 101** | **701** | **2 386** | **7 992** | **5 737** | **3 385** | **900** |
| Age at diagnosis, years ± SD | 46.8±7.7 | 46.8±7.6 | 46.9±7.5 | 47.1±7.6 | 46.9±7.6 | 46.1±7.8 | 46.4±8.0 |
| Cases per 100,000 person-years (95% CI) | 95.2  (93.9-96.5) | 87.4  (81.1-94.1) | 97.2  (93.4-101.2) | 104.0  (101.8-106.3) | 98.3  (95.8-100.9) | 83.8  (81.0-86.7) | 66.8  (62.5-71.3) |
| **Digestive organs, n** | **4 647** | **168** | **443** | **1 628** | **1 244** | **828** | **336** |
| Age at diagnosis, years ± SD | 48.1±9.0 | 48.1±9.3 | 48.8±8.6 | 48.5±8.9 | 48.2±8.9 | 47.2±9.3 | 48.0±9.5 |
| Cases per 100,000 person-years (95% CI) | 20.9  (20.3-21.5) | 20.8  (17.8-24.3) | 17.9  (16.3-19.7) | 21.1  (20.1-22.1) | 21.2  (20.0-22.4) | 20.4  (19.0-21.9) | 24.9  (22.3-27.7) |
| **Female genital organs, n** | **6 839** | **231** | **724** | **2 310** | **1 805** | **1 295** | **474** |
| Age at diagnosis, years ± SD | 43.4±9.4 | 43.2±9.3 | 44.6±9.3 | 43.3±9.3 | 43.3±9.3 | 43.1±9.5 | 43.5±9.5 |
| Cases per 100,000 person-years (95% CI) | 30.7  (30.0-31.5) | 28.7  (25.1-32.6) | 29.4  (27.3-31.6) | 29.9  (28.7-31.2) | 30.8  (29.4-32.3) | 32.0  (30.3-33.8) | 35.1  (32.0-38.4) |
| **Urinary tract, n** | **1 129** | **38** | **117** | **347** | **284** | **249** | **94** |
| Age at diagnosis, years ± SD | 48.3±9.0 | 48.2±9.3 | 47.6±9.6 | 48.7±8.8 | 48.5±9.0 | 47.9±8.7 | 47.7±9.2 |
| Cases per 100,000 person-years (95% CI) | 5.1  (4.8- 5.4) | 4.7  (3.3- 6.5) | 4.7  (3.9- 5.7) | 4.5  (4.0- 5.0) | 4.8  (4.3- 5.4) | 6.1  (5.4- 6.9) | 6.9  (5.6-8.5) |
| **Eye, brain and other parts of central nervous system, n** | **1 673** | **57** | **192** | **580** | **442** | **310** | **92** |
| Age at diagnosis, years ± SD | 43.5±9.5 | 43.9±8.5 | 44.5±9.7 | 43.9±9.6 | 43.5±9.0 | 41.9±9.5 | 43.7±10.0 |
| Cases per 100,000 person-years (95% CI) | 7.5  (7.2- 7.9) | 7.1  (5.4- 9.2) | 7.8  (6.7- 9.0) | 7.5  (6.9- 8.1) | 7.5  (6.8- 8.3) | 7.6  (6.8- 8.05 | 6.8  (5.5-8.3) |
| **Lip, oral cavity and pharynx, n** | **1 146** | **28** | **119** | **399** | **316** | **234** | **50** |
| Age at diagnosis, years ± SD | 45.0±9.5 | 42.5±8.5 | 43.3±9.4 | 45.6±9.5 | 45.3±9.4 | 44.5±9.5 | 45.7±10.2 |
| Cases per 100,000 person-years (95% CI) | 5.1  (4.8-5.4) | 3.5  (2.3-5.0) | 4.8  (4.0-5.8) | 5.2  (4.7-5.7) | 5.4  (4.8-6.0) | 5.8  (5.1-6.6) | 3.7  (2.7-4.9) |
| **Bone and articular cartilage, n** | **385** | **12** | **46** | **127** | **97** | **78** | **25** |
| Age at diagnosis, years ± SD | 42.7±9.4 | 42,1±10.0 | 44.5±7.6 | 43.0±10.0 | 42.0±9.6 | 42.7±9.3 | 41.0±8.4 |
| Cases per 100,000 person-years (95% CI) | 1.7  (1.6-1.9) | 1.5  (0.8-2.6) | 1.9  (1.4-2.5) | 1.6  (1.4-2.0) | 1.7  (1.3-2.0) | 1.9  (1.5-2.4) | 1.8  (1.2-2.7) |
| **Hematopoietic and**  **Lymphatic system, n** | **2 978** | **104** | **320** | **1 000** | **786** | **544** | **224** |
| Age at diagnosis, years ± SD | 43.7±10.1 | 44.2±9.9 | 43.8±10.2 | 44.3±10.1 | 43.7±10.1 | 42.6±9.9 | 43.1±10.5 |
| Cases per 100,000 person-years (95% CI) | 13.4  (12.9-13.9) | 12.9  (10.5-15.6) | 13.0  (11.6-14.5) | 12.9  (12.1-13.8) | 13.4  (12.5-14.4) | 13.4  (12.3-14.6) | 16.6  (14.5-18.9) |
| **Thyroid and other endocrine glands, n** | **1 741** | **55** | **197** | **579** | **460** | **326** | **124** |
| Age at diagnosis, years ± SD | 40.6±8.8 | 40.1±10.0 | 40.7±9.1 | 40.9±8.8 | 40.5±8.8 | 40.4±8.4 | 39.8±9.2 |
| Cases per 100,000 person-years (95% CI) | 7.8  (7.4-8.2) | 6.8  (5.1-8.9) | 8.0  (6.9-9.2) | 7.5  (6.9-8.1) | 7.8  (7.1-8.6) | 8.0  (7.2-9.0) | 9.2  (7.6-10.9) |
| **Mesothelial and soft tissue, n** | **1 374** | **40** | **136** | **465** | **372** | **257** | **105** |
| Age at diagnosis, years ± SD | 44.2±9.7 | 43.2±9.9 | 44.6±9.8 | 45.0±9.6 | 44.2±9.9 | 40.3±9.6 | 43.8±9.6 |
| Cases per 100,000 person-years (95% CI) | 6.2  (5.8- 6.5) | 5.0  (3.5- 6.8) | 5.5  (4.6- 6.5) | 6.0  (5.5- 6.6) | 6.3  (5.7- 7.0) | 6.3  (5.6- 7.2) | 7.8  (6.3-9.4) |
| **Melanoma and other malignant neoplasms of skin, n** | **19 497** | **677** | **2 491** | **7 682** | **4 965** | **2 957** | **725** |
| Age at diagnosis, years ± SD | 47.3±8.5 | 48.0±8.2 | 47.9±8.4 | 47.8±8.4 | 47.1±8.4 | 46.2±8.6 | 45.1±9.0 |
| Cases per 100,000 person-years (95% CI) | 87.8  (86.6-89.1) | 84.3  (78.1-90.9) | 101.4  (97.4-105.4) | 99.8  (97.6-102.1) | 84.9  (82.5-87.3) | 73.1  (70.5-75.8) | 53.7  (49.9-57.8) |

Data presented as event count (n) and event rate (events/ 100000 years (95% CI)). CI; confidence interval, IQR; interquartile range, SD; standard deviation

**Supplementary** **Figure 1**. Flowchart of the study population

Excluded: BMI<15 kg/m^2^ or > 60 kg/m^2^

***n=*1 077**

>200

## ***n=* 1 386 725**

## **Total pregnancies**

## ***n=* 1 393 346**

Excluded: cancer before baseline

***n=*5 174**

Excluded: height<140cm or > 200 cm

***n=*370**

>200

**Supplementary Figure 2.** Hazard ratios of cancer forms in women aged 18-45 years by body mass index categories, exclude individuals with diagnosis of cancer the first year after the registration.


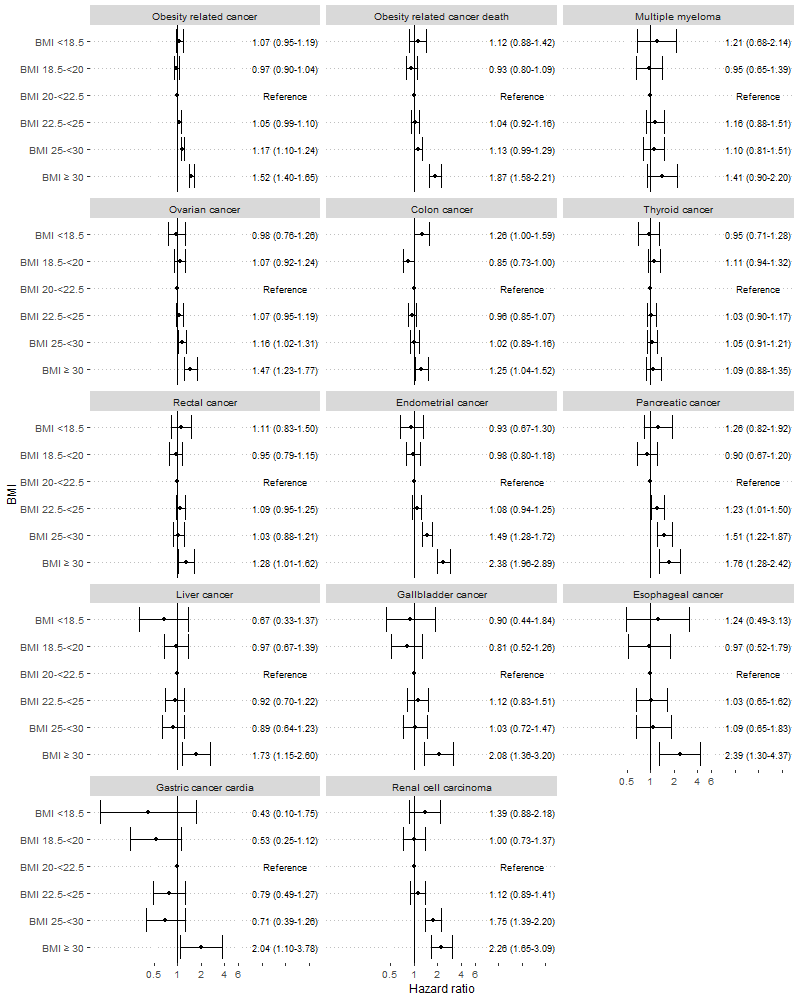


**Supplementary Figure 3**. Risk of cancer by organ system in women aged 18-45 years by body mass index (BMI) categories, expressed as hazard ratios with 95% confidence intervals. Hazard ratios for linear increase in BMI was calculated for a 5-unit BMI increase for BMI above 20, test for nonlinearity was performed by comparing the log-likelihood of models with BMI as a linear term and as a spline.

**
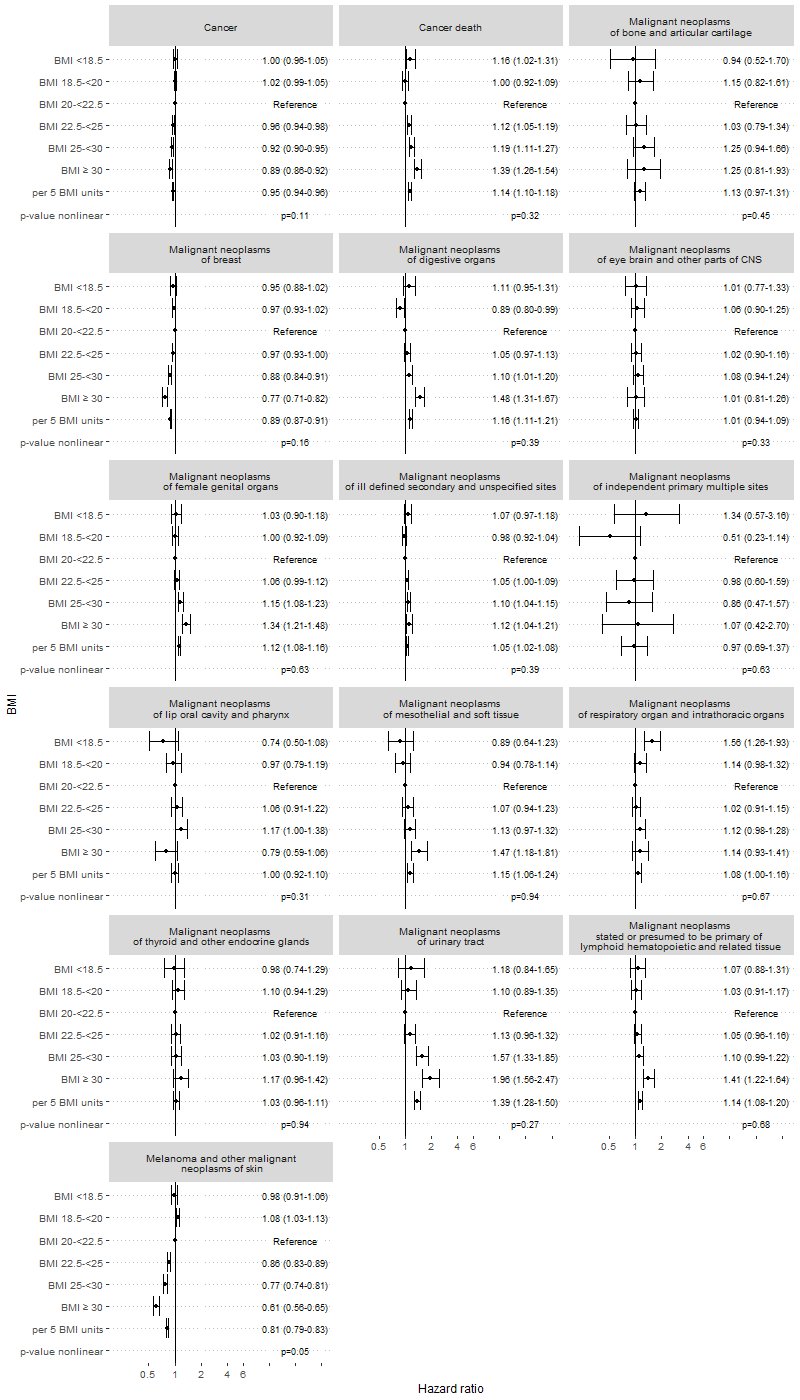
**

**Supplementary Figure 4**. Spline model on the effect of body mass index on the risk for obesity-related cancer incidence and mortality, and breast cancer (Model 1 adjusted for year of pregnancy and parity).


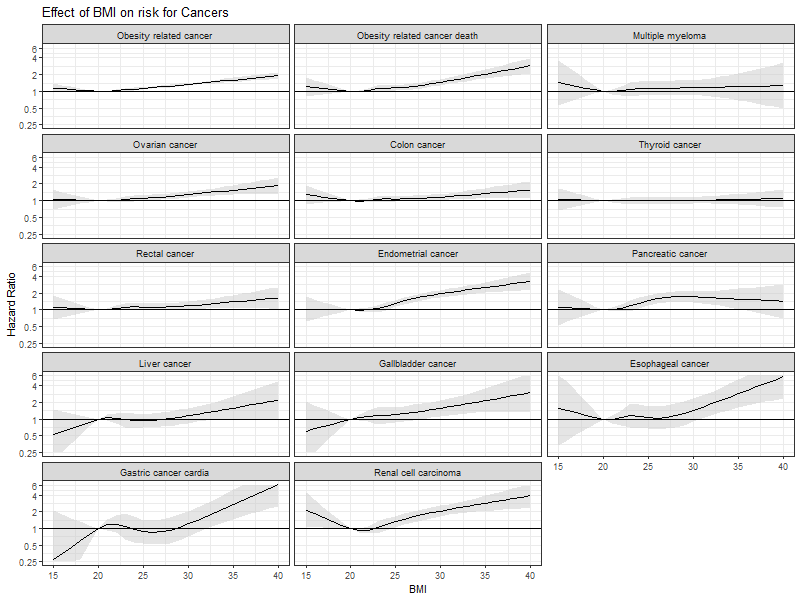


**Supplementary Figure 5.** Spline model on the effect of body mass index on the risk for obesity-related cancer incidence and mortality, and breast cancer (Model 2 adjusted for year of pregnancy, parity, and for diabetes and hypertension at baseline).


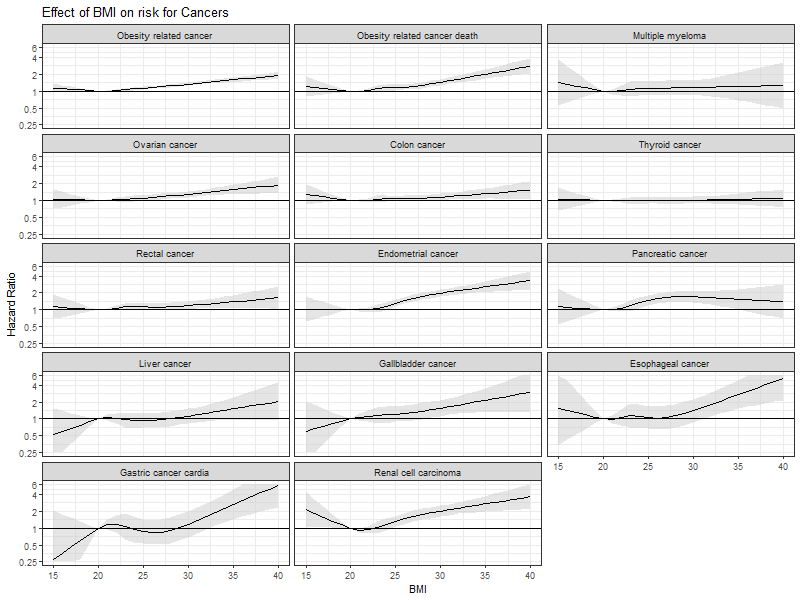

Supplement: Supplementary file 1 — Supplementary Information. [file 41598_2024_56899_MOESM1_ESM.docx]
